# Hydrogen Sulfide and Cellular Redox Homeostasis

**DOI:** 10.1155/2016/6043038

**Published:** 2016-01-05

**Authors:** Zhi-Zhong Xie, Yang Liu, Jin-Song Bian

**Affiliations:** ^1^Institute of Pharmacy & Pharmacology, University of South China, Hengyang, Hunan 421001, China; ^2^Hunan Province Cooperative Innovation Center for Molecular Target New Drug Study, Hengyang, Hunan 421001, China; ^3^Department of Pharmacology, Yong Loo Lin School of Medicine, National University of Singapore, Singapore 117597

## Abstract

Intracellular redox imbalance is mainly caused by overproduction of reactive oxygen species (ROS) or weakness of the natural antioxidant defense system. It is involved in the pathophysiology of a wide array of human diseases. Hydrogen sulfide (H_2_S) is now recognized as the third “gasotransmitters” and proved to exert a wide range of physiological and cytoprotective functions in the biological systems. Among these functions, the role of H_2_S in oxidative stress has been one of the main focuses over years. However, the underlying mechanisms for the antioxidant effect of H_2_S are still poorly comprehended. This review presents an overview of the current understanding of H_2_S specially focusing on the new understanding and mechanisms of the antioxidant effects of H_2_S based on recent reports. Both inhibition of ROS generation and stimulation of antioxidants are discussed. H_2_S-induced S-sulfhydration of key proteins (e.g., p66Shc and Keap1) is also one of the focuses of this review.

## 1. Introduction

In 1777, a young Swedish apothecary, Carl Wilhelm Scheele, treated ferrous sulfide with a mineral acid and noted a colorless gas with a characteristic odor of rotten eggs. He described it as “sulfuretted hydrogen.” The notoriety of hydrogen sulfide (H_2_S) had been considered as a toxic gas for several hundreds of years. The Permissible Exposure Limit (PEL) of H_2_S is 10 ppm and sudden exposure to >400 ppm can cause rapid death. The biological effects of H_2_S in physiological condition began around the turn of the 20th century. H_2_S is now recognized as the third “gasotransmitter” along with nitric oxide (NO) and carbon monoxide (CO) [1]. The desulfhydration of cysteine is considered as the major source of H_2_S in mammals. This process is catalyzed by cystathionine *β*-synthase (CBS) and cystathionine *γ*-lyase (CSE), two pyridoxal-5′-phosphate- (PLP-) dependent enzymes. CBS is primarily expressed in various regions of the brain and is essential to the production of H_2_S in the central nervous system [2–4], whereas CSE is mainly expressed in the cardiovascular system [5, 6]. Recently, 3-mercaptopyruvate sulfurtransferase (3-MST) was reported as the third enzyme for H_2_S production, which is localized to mitochondria and nerve endings [7, 8]. We and others proved that H_2_S exerted a wide range of biological functions including neuroprotection [9, 10], cardioprotection [11, 12], antihypertension [13], and osteoblastic protection [14]. The antioxidant effect of H_2_S has been most extensively investigated and was thought as the major mechanism underlying the effects of H_2_S. Here, we summarize the existing knowledge about the antioxidant effect of H_2_S, highlighting recent advances in our understanding of the ability of H_2_S to neutralize reactive oxygen species (ROS) in vivo.

## 2. Free Radical, Oxidative Stress, and Cellular Antioxidant Defenses

### 2.1. Free Radical and Oxidative Stress

A free radical is an unstable chemical species that contains one or more unpaired electrons in its outer orbital. In organisms, the highly reactive free radicals formed from metabolism might donate their unpaired electron to another molecule or pull an electron off a neighboring molecule. The term oxidative stress has been proposed indicating a disturbance in the equilibrium status of oxidant/antioxidant systems with a progressive accumulation of ROS in intact cells. ROS are short-lived and highly chemically reactive. At low concentrations, ROS serve as cellular signaling molecules [15]. However, at high concentrations, ROS may cause both beneficial and unbeneficial effects. In the late case, ROS may not only kill invading pathogens and microbes but also damage the components of the cell, including proteins, lipids, carbohydrates, and DNA [16]. Overwhelming evidence indicates that oxidative stress is involved in the pathophysiology of the wide array of human diseases, including cancer [17], cardiovascular disease [18], AIDS [19], diabetes mellitus [20], and neurodegenerative disorders such as aging, Parkinson's disease, and Alzheimer's disease [21, 22].

In human body, more than 95% free radicals belong to oxygen free radicals. Recent studies suggest that oxygen-free radicals play an essential role in the control of cell functions and signal transmission [23, 24]. The common oxygen free radicals consist of superoxide anion (^•^O_2_^−^), hydroxyl radical (HO^•^), perhydroxyl radical (HO_2_^•^), alkoxyl radical (RO^•^), alkyl peroxide radical (ROO^•^), and so on (Table 1). Among them, ^•^O_2_^−^ is very unstable and able to react spontaneously with itself producing hydrogen peroxide (H_2_O_2_) and molecular oxygen (O_2_) [25]. ^•^O_2_^−^ is the starter of chain reaction of oxygen free radicals. HO^•^ is the most reactive oxygen free radical and can react with any biological molecule [26]. HO_2_^•^ is the protonated form of superoxide anion and exhibits higher reactivity than superoxide anion [27]. In addition, other reactive oxygen metabolites such as H_2_O_2_ and the singlet oxygen (^1^O_2_) can also be regarded as oxygen free radicals, although they are not true free radical species. H_2_O_2_ may cross the biological membranes and is one of the origins of highly reactive HO^•^ [28]. The singlet oxygen (^1^O_2_) also has higher reactivity [29] and can be formed directly by illumination (*hv*) from molecular oxygen.

### 2.2. The Sources of ROS

ROS are widespread in living organisms. Actually, they are being continuously produced in vivo and many of them are necessary to carry out certain cellular and biological reactions [23]. When they were overproduced, cellular damage may happen [30, 31]. The origin of oxygen-free radicals may be generated exogenously or endogenously.

**Figure 1:**
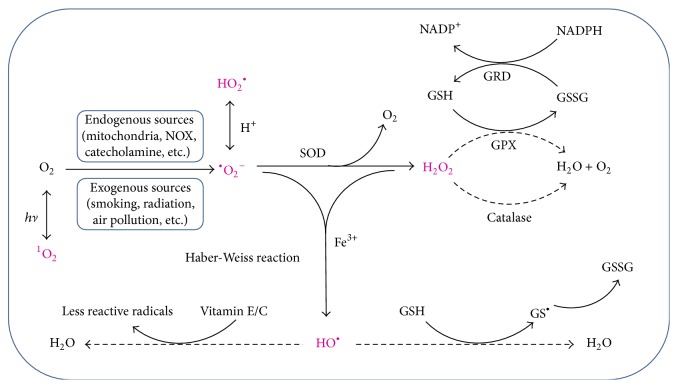
The main ROS generation and elimination pathways. (1) ROS (including ^•^O_2_^−^, HO_2_^•^, HO^•^, ^1^O_2_, and H_2_O_2_) may be generated by exogenous (like smoking, *hν*, air pollution, etc.) and endogenous (like mitochondria, catecholamine, NOX, etc.) stimulating factors. ^•^O_2_^−^ can further react with H_2_O_2_ to generate HO^•^ through the Haber-Weiss reaction in the presence of ferric irons (shown as solid line arrows). (2) Excessive ^•^O_2_^−^ is eliminated by SOD by catalyzing the dismutation of ^•^O_2_^−^ to H_2_O_2_ and O_2_. H_2_O_2_ can be further removed by the catalysis of CAT or GPx. The catalysis of GPx needs GSH as its cosubstrate and GSH is oxidized to GSSG. GSSG can be reduced to GSH again by GR utilizing NADPH. GSH can also react with oxygen free radical directly and form the thiyl radical (GS^•^) and later GSSG. Vitamin E and vitamin C may react with oxygen free radical and form less reactive radicals (shown as dotted line arrows). NADPH: nicotinamide-adenine dinucleotide phosphate; NOX: NADPH oxidase; XO: xanthine oxidase; SOD: superoxide dismutase; GSH: glutathione; GSSG: glutathione disulfide; GPx: glutathione peroxidase; GR: glutathione reductase.

Exogenous sources are mainly generated by some stimulating factors. These include smoking, alcohol, certain drugs, air pollution, ionizing radiation, and hyperbaric oxygen poisoning. Compared with exogenous sources, endogenous sources play more important roles in the form of oxygen free radicals. Endogenous activities are the main sources of oxygen-free radicals in living organisms. The main endogenous sources are listed below (Figure 1, solid line arrows).

*(i) Mitochondrial Electron Transport*. The oxygen-free radical is the by-products of cellular metabolism. Under normal physiological conditions, most oxygen in organisms will acquire four electrons and four protons and reduce to form water by the cytochrome c oxidase from electron transport system of mitochondrial. In this procedure, no oxygen-free radical will form at last. However, if the molecular oxygen undergos sequential univalent reduction, highly reactive ^•^O_2_^−^, HO^•^, and H_2_O_2_ would be formed [32]. Mitochondria are the major source of intracellular ROS. As the terminal electron acceptor of respiration, more than 90% oxygen is used to produce ATP in mitochondria and about 2% of the oxygen is transformed into ROS as respiratory chain by-products [33].

**Figure 2:**
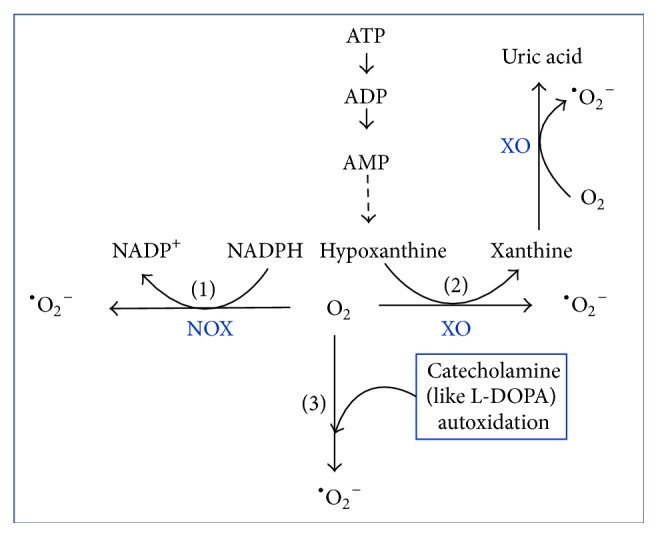
Endogenous superoxide anion (^•^O_2_^−^) formation pathways. (1) NOX catalyzes the one-electron reduction of oxygen to produce ^•^O_2_^−^ by utilizing NADPH as an electron donor. (2)  ^•^O_2_^−^ formation in the process of the XO-catalyzed conversion of hypoxanthine into xanthine or xanthine into uric acid. (3)  ^•^O_2_^−^ formation within catecholamine autooxidation.

*(ii) The Increase of Xanthine Oxidase (XO)*. There are about 10% of xanthine oxidases (XO) and 90% of xanthine dehydrogenase (XD) in endothelial cells. The xanthine dehydrogenase (XD) will be converted into xanthine oxidase (XO) during ischaemia [34]. In this condition, the adenosine triphosphate (ATP) could not release energy. Instead, it will be degraded into adenosine diphosphate (ADP), adenosine monophosphate (AMP), and hypoxanthine gradually. Upon reperfusion of the ischemic tissue, increased xanthine oxidase (XO) will convert the increased hypoxanthine to xanthine and then convert the formed xanthine to uric acid by using oxygen as electron acceptor. Oxygen is reduced and produced ^•^O_2_^−^, HO^•^, and H_2_O_2_ finally [35] (Figure 2).

*(iii) The Increase of Catecholamine*. Sympathetic adrenomedullary system is an important stress regulation system in our bodies. Catecholamine produced by this system under an external stimulus has an important role in the adjustment of metabolism. Catecholamine can also be converted to oxygen-free radicals by autooxidation [36] (Figure 2). It is worth noting that too much catecholamine and their oxidation products, especially the superoxide anion radicals, will cause damage to the body.

*(iv) NADPH Oxidase*. Nicotinamide-adenine dinucleotide phosphate (NADPH) oxidase (NOX) is another important enzyme for intracellular ROS generation. It is mainly distributed in the plasma membrane surface of phagocyte and catalyzes the one-electron reduction of oxygen to produce superoxide-free radical by utilizing NADPH as an electron donor (Figure 2). The NOX system is dormant in normal, but it can be activated by some stimulating factors, such as leukotriene, endotoxin, complement, and calcium ion [37, 38]. Thereby, more oxygen (O_2_) will be quickly reduced to ^•^O_2_^−^ and H_2_O_2_. HO^•^ will be also formed by further metabolism [39].

*(v) Catalysis of Transitional Metals*. The transitional metals, such as iron and copper, can change their valence by donating an electron and thus catalyze the Haber-Weiss reaction [28] as shown in (1). In the presence of superoxide anions, ferritin-bound ferric iron in cells usually can be liberated as ferrous form, thereby increasing the amount of iron [40]. The generated iron can form HO^•^ in the presence of hydrogen peroxide. This is the main source of hydroxyl radicals.

(1) O2•−+H2O2→Fe3+O2+HO−+HO•

In addition, the metabolism of arachidonic acid by cyclooxygenase [41, 42] or lipoxygenases [43, 44], cytochromes P450 of the microsomal electron transport system [45, 46], may also produce oxygen-free radicals.

### 2.3. Cellular Defenses of ROS

In living organisms, ROS are continuously produced because of the reduction of molecular oxygen. Although free radicals play an important role in some physiological reactions, such as cell signal transduction and regulation of muscle tone [23, 40], excessive free radicals would cause damage to the lipids, proteins, and DNA and give rise to cellular and metabolic disturbance [30]. There are enzymes and chemical scavengers that could be used to remove excessive oxygen-free radicals formed in a living body [47].

Superoxide dismutase (SOD) is a common antioxidant enzyme which contains copper, zinc, and manganese as cofactors [48]. SOD can catalyze the dismutation of ^•^O_2_^−^ to molecular oxygen (O_2_) and the lesser active species H_2_O_2_ at a higher rate than the spontaneous dismutation of ^•^O_2_^−^. The formed H_2_O_2_ will be further decomposed to H_2_O and O_2_ or be used to form HO^•^ through the Haber-Weiss reaction, as shown in (1), and reduced to H_2_O finally (Figure 1, dotted line arrows).

Catalase (CAT) is another antioxidant enzyme that is widely distributed in tissues [49]. It could catalyze the degradation of H_2_O_2_ directly to water and prevent the secondary generation of other intermediate radicals. In addition, selenium-containing glutathione peroxidase (GPx) could also catalyze the reduction of H_2_O_2_ [50]. This reaction needs reduced glutathione (GSH) as cosubstrate and GSH will be oxidized to oxidized glutathione (GSSG). GSSG could also be reduced to GSH again by glutathione reductase (GR) utilizing NADPH.

There are also some nonenzymatic chemical antioxidants that play an important role in antioxidant, included glutathione (GSH), *α*-tocopherol (vitamin E), and ascorbic acid (vitamin C) [51]. As mentioned before, GSH can act as a cosubstrate in the reduction of H_2_O_2_ by GPx. GSH could also react with oxygen-free radical directly and form the thiyl radical and later GSSG [52]. Like GSH, vitamins E and C could also reduce oxygen-free radicals [53]. They would trap hydroxyl radicals and other reactive radicals and thus break radical chain reactions and form new less reactive radicals. These new formed radicals themselves could not be removed or make further conversion. Only with the help of GPx and other biological molecules, these new radicals can be transformed to nonreactive substances. So, excess supplement of vitamins and other chemical antioxidants is not necessary. Excess levels of vitamins cannot replace the position of enzymes in organisms [54]. On the contrary, excess chemical antioxidants may produce excess less reactive radicals by reacting with oxygen radicals. These excess radicals may cause damage to the body. Actually, chemical antioxidants usually go into effect with the cooperation of antioxidant enzymes [55].

## 3. Mechanisms for the Regulatory Effect of H_2_S on ROS In Vivo

### 3.1. Quenching Free Radicals as a Chemical Reductant

At 37°C and pH 7.4, more than 80% of H_2_S molecules dissolve in surface waters and dissociate into H^+^, HS^−^, and S^2−^ ions. HS^−^ is powerful one-electron chemical reluctant and presents a remarkable capacity to scavenge ROS. In addition, H_2_S itself has also been recognized to be a poor reducing agent, which can react directly with and quenches the superoxide anion (O_2_^−^) [56, 57] and NO-free radicals like peroxynitrite [58] as well as other ROS in vitro. However, it should be noted that the physiological concentration of H_2_S in vivo is believed to be at the submicromolar range [59, 60] and such low concentration of H_2_S is not paralleled with its antioxidant effect. Moreover, in our previous work, NaHS pretreatment significantly inhibited H_2_O_2_-induced (50 *μ*M, 2 h) mitochondrial ROS generation and protected human neuroblastoma SH-SY5Y cells against H_2_O_2_-induced injury even when it had been washed out before H_2_O_2_ administration. Similar effects were also found in MC3T3-E1 osteoblastic cells, and this antioxidant effect of H_2_S lasted for at least 18 h [14]. These results indicated that other mechanisms besides chemical reductant exist in the antioxidant effect of H_2_S. We speculate that H_2_S might act as a trigger which will be retired after starting the process of antioxidant action.

### 3.2. Scavenging Free Radicals In Vivo via Nonenzymatic Antioxidants

As we mentioned before, ROS is counterbalanced in the body by a net of antioxidants, including enzymatic and nonenzymatic antioxidants. GSH and thioredoxin (Trx-1) are two biologically important nonenzymatic antioxidants in animal cells and attracted increasing attention as cellular protectants against oxidative stress in vivo.

#### 3.2.1. H_2_S Increases Intracellular Reduced Glutathione (GSH)

**Figure 3:**
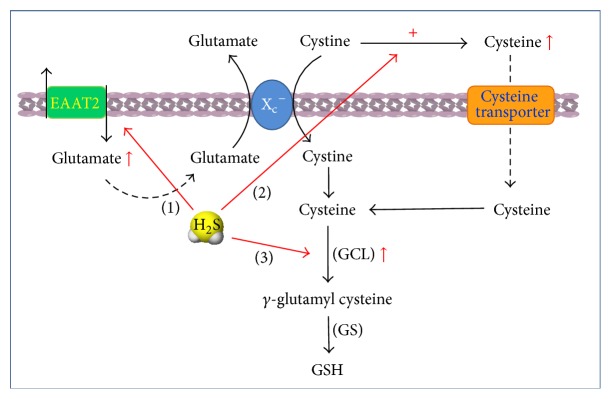
H_2_S increases intracellular GSH synthesis. Cellular GSH is mainly synthesized from cysteine. (1) H_2_S increases EAAT2-mediated glutamate uptake, which thereby increases cystine transportation through cystine/glutamate antiport system (X_c_^−^). (2) Intracellular H_2_S is released into extracellular space and reduces cystine into cysteine, which would be efficiently imported into cells through a cysteine transporter distinct from system X_c_^−^. These two pathways provide more substrate to produce GSH. (3) H_2_S increases glutamate cysteine ligase (GCL) expression and promotes GSH synthesis.

GSH, a tripeptide consisting of cysteine, glutamate, and glycine, is a major antioxidant in the cellular defense against oxidative stress and a decreased GSH/GSSG ratio is usually taken as indicating oxidative stress. In cells, GSH is synthesized from cysteine. There are 2 cysteine forms, oxidized form cystine and reductive form cysteine. Because of its redox instability, extracellular cysteine is mostly present in cystine, which can be transported into cells through cystine/glutamate antiport system X_c_^−^, then reduced to cysteine, and used for GSH synthesis [61]. Glutamate is the key inhibitor of the system X_c_^−^. Our previous study showed that NaHS at 100 *μ*M promoted [^3^H]glutamate uptake in astrocytes via enhancing the trafficking of glial glutamate transporter GLT-1 (also named the excitatory amino acid transporters-2, EAAT2), enhanced cystine transport, and increased intracellular GSH synthesis finally [62] (Figure 3).

Studies from other laboratories have also proven that H_2_S preserves the cellular GSH status and provides protection against oxidative damage in brain [63, 64], spinal cord [65], heart [66, 67], lung [68], kidney [69, 70], liver [71], gastrointestinal tract [72, 73], and so forth. Recently, Kimura et al. showed a different mechanism for H_2_S on intracellular GSH production. They reported that H_2_S produced in cells may be released into extracellular space and reduces cystine into cysteine, which thereby would be efficiently imported into cells through a cysteine transporter distinct from system X_c_^−^ and used for GSH synthesis [74] (Figure 3). Meanwhile, Jain et al. also demonstrated that H_2_S increased intracellular GSH production by upregulating the glutamate-cysteine ligase catalytic subunit (GCLC) and glutamate-cysteine ligase modifier subunit (GCLM) [75] (Figure 3).

#### 3.2.2. H_2_S Increases Intracellular Trx-1

Classic thioredoxin (Trx-1) is a small (12 kDa) ubiquitous molecule containing a characteristic Cys-Gly-Pro-Cys motif and the oxidation-reduction of Trx-1 occurs at its two cysteine residues. It was reported that Trx-1 exerts extracellular and intracellular multifunctions in cell proliferation [76], apoptosis [77], and gene expressions [78]. Moreover, Trx-1 was also shown to scavenge ROS and protect cells against oxidative stress. Trx-1 reduces hydrogen peroxide via peroxiredoxin (Prx) and oxidized Trx-1 is reduced by thioredoxin reductase [79]. Antioxidant effects of Trx-1 can also be mediated indirectly (for more details, see [79]).

In 2008, Jha et al. reported that H_2_S protected murine liver against ischemia-reperfusion (I/R) injury through upregulation of intracellular Trx-1 along with an increase in hepatic tissue GSH/GSSG ratio [71]. Trx-1 was also proved to mediate the cardioprotective effects of H_2_S in the setting of ischemic-induced heart failure by Nicholson et al. [80]. They demonstrated that Na_2_S treatment not only significantly increased the gene and protein expression of Trx-1 but also efficiently improved cardiac dilatation, dysfunction, and hypertrophy in the ischemic heart failure mice. Moreover, they generated transgenic mice with a cardiac-specific overexpression of a dominant negative mutant of Trx-1 and found the cardioprotective effects of Na_2_S were Trx-1 dependent.

#### 3.2.3. Potential Mechanisms of H_2_S on Nonenzymatic Antioxidants Production

Despite the potential role of H_2_S in the cellular antioxidant defense, studies on its antioxidant mechanism have been exceptionally limited. Recently, increasing evidence revealed that Nrf2 participated in the antioxidant effect of H_2_S by promoting cellular antioxidant gene expression.

**Figure 4:**
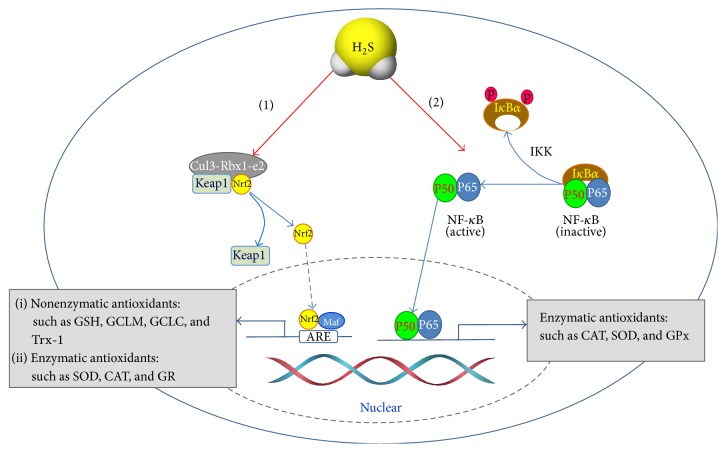
Effect of H_2_S on intracellular enzymatic and nonenzymatic antioxidant production. (1) H_2_S activates Nrf2, which translocates to nuclear, binds to ARE, and upregulates enzymatic and nonenzymatic antioxidant production. (2) H_2_S stimulates NF-*κ*B signaling, which further upregulates the expression of numerous genes including SOD, CAT, and GPx.

Nuclear factor (erythroid-derived 2)-like 2, also known as nuclear factor-erythroid 2 (NF-E2) related factor 2 (Nrf2), is a transcription factor that regulates a wide variety gene expression. Nrf2 is found mostly in the cytoplasm as an inactive complex with Kelch-like ECH-associated protein 1 (Keap1) [81]. Under oxidative stressed conditions, Keap1 undergoes ubiquitination and promotes Nrf2 translocation to the nucleus, in which Nrf2 binds to promoters containing the antioxidant response element (ARE) sequence and inducing ARE-dependent gene expression [82]. ARE is a* cis*-acting regulatory element, which is found in promoter region of certain genes, such as Trx-1 [83], glutathione reductase [84], and thioredoxin-interacting protein (Txnip) [85]. Nrf2 can suppress the basal expression of Txnip, which binds redox-active cysteine residues of Trx-1 and inhibit its antioxidant function [85]. Nrf2 can also increase both expression and activity of glutathione reductase, which, as we mentioned above, promotes oxidized GSH recycle back to reduced GSH and increases GSH/GSSG ratio [86]. On the other hand, it was reported that H_2_S can S-sulfhydrated Keap1 at cysteine-151, which causes a conformational change in Keap1 and thereby leads to Nrf2 dissociation from Keap1. The activated Nrf2 nuclear finally translocates to nuclear and promotes antioxidant gene transcription, such as GCLM, GCLC, and glutathione reductase (GR) [87]. In addition, Calvert et al. also demonstrated that H_2_S increased the expression of Trx-1 and mediated cardioprotection through Nrf2 signaling [83]. Taken together, these results demonstrate that Nrf2 is the potential endogenous cardioprotective signal in the process of cellular nonenzymatic antioxidant generation induced by H_2_S (Figure 4).

### 3.3. Scavenging Free Radicals In Vivo via Enzymatic Antioxidants

Another major mechanism for cells to maintain redox equilibrium is based on the clearance ability processed by cellular antioxidant enzymes. Superoxide dismutase (SOD), CAT, and GPx are three main antioxidant enzymes that defend against oxidative damage in vivo. There are three isoforms of mammalian SOD: the cytosolic copper/zinc-containing SOD (Cu/ZnSOD, SOD-1), the mitochondrial manganese-containing SOD (MnSOD, SOD-2), and the extracellular SOD (ecSOD, SOD-3). SOD catalyzes the dismutation of ^•^O_2_^−^ into H_2_O_2_, while CAT reacts with H_2_O_2_ to form water and molecular oxygen, and GPx detoxifies H_2_O_2_ in the presence of GSH, producing H_2_O and GSSG which is recycled to GSH by glutathione reductase in an NADPH-consuming process [88] (Figure 1, dotted line arrows). In 1995, Searcy et al. reported that H_2_S is a genuine substrate of SOD and can bind at the catalytic Cu center of SOD [89]. The binding of HS^−^ to SOD is very quick and the rate constant for binding is >10^7^ M^−1^ S^−1^. When sulfide combined with SOD, there was a synergistic increase in the rate of superoxide anion scavenging. The *K*_*m*_ measured by the pyrogallol technique is ~80 *μ*M HS^−^ [89]. Recent studies also demonstrated that H_2_S could ameliorate cellular oxidative stress by improving activities of CAT [66, 90–92] and GPx [92–95].

The signal transduction pathways for H_2_S to promote endogenous enzymatic antioxidant defense are much less understood. NF-*κ*B is a family of transcription factors and plays a pivotal role in inflammation. H_2_S was reported to attenuate inflammation via inhibition of NF-*κ*B activation, which is associated with an array of diseases, such as hypoxia-induced neurotoxicity [96], cerebral ischemia [97], kidney injury [98], pulmonary fibrosis [99], and acute pancreatitis [100]. However, as a redox-sensitive transcription factor, NF-*κ*B has also been considered as the most important factor on regulation of cellular antioxidant enzymes and was reported to be upregulated by H_2_S via substance P [101, 102]. Analyzing the gene sequences of mouse GPx and CAT, Zhou et al. [103] revealed the existence of binding sites for NF-*κ*B at position -283 in the GPx gene and at the -227 and -242 in the CAT gene. Additionally, SOD was also proved to have binding site for NF-*κ*B in its 5′-flanking region and the DNA binding activity of NF-*κ*B was induced in response to oxidative stress [104]. Taken together, these observations suggest that NF-*κ*B mediated signaling pathway is most likely attributable to the augmentation of endogenous antioxidant capacity of H_2_S in response to oxidative stress (Figure 4).

In addition to the activation of NF-*κ*B, Nrf2 signaling cascade maybe another rational that accounts for the antioxidant effect of H_2_S. Dreger et al. [105] identified that an ARE element existed in the SOD1 and CAT promoter, which is not only essential but also sufficient for transcriptional regulation. In their study, antioxidative enzymes in cardiac myocytes were induced via Nrf2-dependent transcriptional activation of ARE sites. On the other hand, diallyl sulfide (DAS), a kind of sulfur-containing compound, was demonstrated to cause a significant increase in the activities of SOD, CAT, GPx, GR, glutathione-S-transferase (GST), and quinone reductase (QR) in rat kidney through the activation of Nrf2 to protect the cell against oxidative stress [106]. This indicates a possible role of H_2_S in ROS-interacting enzymes synthesis. However, there is no direct report to link the effect of H_2_S on Nrf2 signal pathway to Nrf2-induced antioxidative enzymes synthesis at present and further investigations are needed in future.

### 3.4. Inhibitory Effect on Mitochondrial Free Radicals Production

Besides the capacity of cellular antioxidant defense, sequential overproduction of ROS is another vital factor in response to oxidative stress. Mitochondria is the major source of intracellular ROS and leak from the electron transfer chain is thought to be the main route [107]. Mounting evidence shows that p66Shc plays predominant roles in mitochondrial redox signaling and its phosphorylation at serine-36 acts as a switch on mitochondrial ROS production [108, 109].

**Figure 5:**
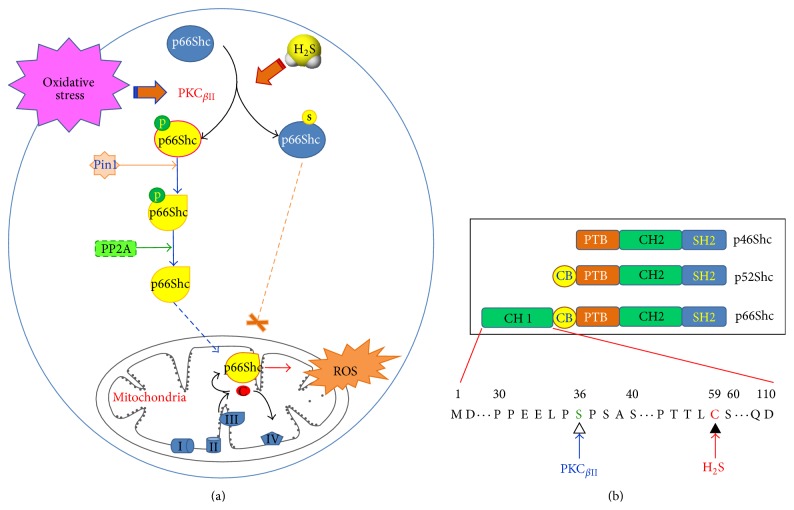
Proposed model for the effect of H_2_S on p66Shc mediated mitochondrial ROS generation. (a) Showing the effect of H_2_S. p66Shc is activated by PKC_*β*II_-dependent phosphorylation at serine-36 in the N-terminal CH2 domain. The phosphorylated p66Shc can be isomerized by Pin1 and dephosphorylated by PP2A. This, in turn, induces mitochondrial translocation of p66Shc and ROS production. H_2_S sulfhydrates p66Shc at cysteine-59, which locates in the same CH2 domain besides serine-36, disrupts the association between PKC_*β*II_ and p66Shc, inhibits PKC_*β*II_-mediated p66Shc phosphorylation, and decreases mitochondria ROS generation finally. (b) Showing the structure of p66Shc and the conserved serine (Ser-36) and cysteine (Cys-59) residues in the CH2 domain.

p66Shc is a 66 kD Src homologous-collagen homologue (Shc) adaptor protein, which is encoded by the* shc1* gene and belongs to the ShcA family. There are two other Shc family members, p46Shc and p52Shc, and all these 3 isoforms share three common functionally identical domains: the C-terminal Src homology 2 domain (SH2), the central collagen homology domain (CH1), and the N-terminal phosphor-tyrosine-binding domain (PTB) [110]. Different from the other two isoforms, p66Shc has an additional N-terminal CH2 domain which contains a critical serine residue at the position 36 (Ser-36) and shows different functions from p46Shc and p52Shc. It was proved that p66Shc has a negative influence on the Ras-mediated signaling pathway [111] but is involved in mitochondrial redox signaling. In response to oxidative stress (UV exposure or H_2_O_2_ treatment), p66Shc is phosphorylated by protein kinase C-*β*II (PKC_*β*II_) at Ser-36. The activated p66Shc is then isomerized by the prolyl isomerase Pin1 and dephosphorylated by phosphatase A2 (PP2A) and finally translocates to mitochondria, where it binds to cytochrome c and transfers electrons from cytochrome c to molecular oxygen to product ROS [112, 113] (Figure 5). Migliaccio et al. reported that p66Shc^−/−^ mice have a 30% increase in the life span [114]. Consistent with this report, Tomilov et al. also demonstrated that macrophages from p66Shc^−/−^ mice appeared to have defect in the activation of the NADPH oxidase and therefore less superoxide production was observed [115].

Recently, our group demonstrated for the first time that H_2_S may inhibit mitochondrial ROS production via a p66Shc-dependent signal transduction. Protein S-sulfhydration had been proposed to emerge as a major functional alteration of proteins, such as the potassium channels (like KATP, IKca, and SKca) [116], PTP1B [117], NF-*κ*B [118], and Keap1 [87]. We proved that H_2_S sulfhydrated p66Shc at cysteine-59, which resides in the proximity to the phosphorylation sites serine-36. S-sulfhydration of p66Shc further impaired the association of PKC_*β*II_ and p66Shc, attenuated H_2_O_2_-induced p66Shc phosphorylation, and reduced mitochondrial ROS generation [119]. This new finding provides new insights and clues to better understand the important role of the H_2_S in oxidative stress and oxidative stress related disease (Figure 5).

## 4. Challenges and Conclusions

The antioxidant activity of H_2_S discussed in this review illuminated the biochemical mechanisms of H_2_S on cellular redox homeostasis. However, the effects of H_2_S on redox status are highly divergent. H_2_S was also reported as a powerful prooxidant, which kills cancer cells in a ROS-dependent manner [57]. It was believed that the Janus-faced molecule serves as an antioxidant or a prooxidant depending on its local concentrations. At lower concentrations, H_2_S exerts beneficial effects like protective effects in the cardiovascular system as we mentioned before, while at higher concentrations, H_2_S exhibits a variety of deleterious/cytotoxic effects (for more details, see [120]).

It should also be noted that the concentration- and time-dependent effects of H_2_S are very complicated. H_2_S was reported to display opposite effects at different concentrations/periods. GYY4137, a slow-releasing H_2_S donor, yielded very low concentrations of H_2_S and was proved to kill cancer cells. NaHS, which releases higher concentrations of H_2_S in short period, however, only exhibited weaker anticanner effect [121]. This may imply that both H_2_S releasing speed and amount are important for its therapeutic effects. Therefore, the biological functions of H_2_S should be studied in different pathological situations with varied concentrations and treatment periods. Endogenous H_2_S generating enzyme activities should also be taken into consideration, as they may be activated/inhibited upon cellular oxidative stress.

In summary, we discussed the current understanding of the antioxidant effect of H_2_S in this paper. Obviously, H_2_S does not produce antioxidant effect via a single/simple mechanism. Multiple targets and signaling pathways are involved. H_2_S can stimulate cellular enzymatic or nonenzymatic antioxidants to scavenge free radicals. This may be secondary to a direct effect on antioxidants or an indirect action through activation of various signaling proteins. H_2_S may also inhibit mitochondria ROS production through sulfhydration of p66Shc or membrane/cytosol ROS generation via inhibition of NADPH. To a weak extent, H_2_S also quenches free radicals directly due to its chemical reducing property. Future studies to explore more action sites of H_2_S in different signaling proteins and mechanisms underlying concentration- and time-dependent effects of H_2_S are still warranted.

## Figures and Tables

**Table 1:** The types of common oxygen-free radicals.

Radicals	Chemical formulas	Electron structures
Superoxide anion	^∙^O_2_^−^	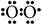
Hydroxyl radical	HO^∙^	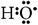
Perhydroxyl radical	HO_2_^∙^	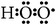
Alkoxyl radical	RO^∙^	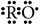
Alkyl peroxide radical	ROO^∙^	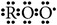
Hydrogen peroxide	H_2_O_2_	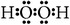
Singlet oxygen	^1^O_2_	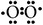

## Abbreviations

3-MST: 3-Mercaptopyruvate sulfurtransferase
ADP: Adenosine diphosphate
AMP: Adenosine monophosphate
ARE: Antioxidant response element
ATP: Adenosine triphosphate
CAT: Catalase
CBS: Cystathionine *β*-synthase
CH1: collagen homology domain 1
CO: carbon monoxide
CSE: Cystathionine *γ*-lyase
DAS: Diallyl sulfide
GCLC: Glutamate-cysteine ligase catalytic subunit
GCLM: Glutamate-cysteine ligase modifier subunit
GPx: Glutathione peroxidase
GR: Glutathione reductase
GSH: Reduced glutathione
GSSG: Oxidized glutathione
GST: Glutathione-S-transferase
I/R: Ischemia-reperfusion
Keap1: Kelch-like ECH-associated protein 1
NO: Nitric oxide
Nrf2: Nuclear factor-erythroid 2 (NF-E2) related factor 2
P.E.L: Permissible Exposure Limit
Pin1: Prolyl isomerase
PP2A: Dephosphorylated by phosphatase A2
Prx: Peroxiredoxin
PTB: The phosphor-tyrosine-binding domain
QR: Quinone reductase
ROS: Reactive oxygen species
Shc: Src homologous-collagen
SOD: Superoxide dismutase
Trx-1: Thioredoxin 1
XD: Xanthine dehydrogenase
XO: Xanthine oxidase.
